# Applications of Nanotechnology in Ruminant Animal Production: Advances, Challenges, and Future Prospects

**DOI:** 10.3390/nano15231773

**Published:** 2025-11-26

**Authors:** Ahmed E. Kholif, Anuoluwapo Anele, Mireille Chahine, Uchenna Y. Anele

**Affiliations:** 1 Department of Animal Sciences, North Carolina Agricultural and Technical State University, Greensboro, NC 27411, USA; uyanele@ncat.edu; 2 Dairy Science Department, National Research Centre, 33 Bohouth St. Dokki, Giza 12622, Egypt; 3 Department of Biology, North Carolina Agricultural and Technical State University, Greensboro, NC 27411, USA; aoanele@ncat.edu; 4 Department of Animal, Veterinary and Food Sciences, University of Idaho, 315 Falls Ave, Twin Falls, ID 83301, USA

**Keywords:** ruminant nutrition, nano-minerals, nano-encapsulation, feed efficiency, methane mitigation, livestock sustainability, nano-sensors

## Abstract

Nanotechnology offers innovative approaches to improve ruminant nutrition by enhancing feed efficiency, nutrient utilization, animal health, and environmental sustainability. This review highlights the use of nano-minerals, nano-encapsulated bioactives, enzyme nano-particles, and nano-sensors to optimize rumen function, digestion, and immunity. Nano-minerals provide high bioavailability at lower doses and may replace antibiotics. Encapsulated compounds like essential oils, probiotics, and vitamins improve rumen fermentation and product quality. Nanotechnology allows precise nutrient delivery through encapsulation, chelation, and nano-packaging without affecting feed sensory properties. Nano-particles are classified as inorganic, organic, or complex nano-structures and are synthesized using physical, chemical, or biological methods. While promising, nanotechnology adoption must address concerns related to safety, environmental impact, and cost. Robust risk assessments and regulatory frameworks are essential. Overall, nanotechnology represents a powerful tool for advancing sustainable and profitable ruminants, and continued multidisciplinary research is needed to fully realize its benefits and ensure its responsible application in animal agriculture.

## 1. Introduction

Ruminants such as cattle, sheep, goats, and buffalo serve as key contributors to global food systems by converting low-quality, fibrous plant materials into nutrient-dense animal. However, the industry is under increasing pressure due to rising feed costs, inefficient nutrient use, environmental impacts—particularly greenhouse gas emissions—and the growing global demand for sustainable animal-source foods. These challenges highlight the urgency for innovative, science-driven approaches to improve productivity while safeguarding environmental and food safety standards.

Nanotechnology, which involves the manipulation of materials at dimensions between 1 and 100 nanometers (10^−9^–10^−7^ m), has gained considerable momentum in agricultural and animal sciences for its unique structural and functional properties [1]. In the context of ruminant nutrition, nano-scale interventions offer new strategies to overcome the limitations of traditional feed additives. Their small particle size and large surface area enhance the solubility, stability, and absorption of nutrients and bioactives in the gastrointestinal tract [2].

Nano-minerals, nano-encapsulated phytochemicals, and enzyme-based nano-particles (NPs) have shown the potential to optimize ruminal digestion, improve feed efficiency, and support better health outcomes [3,4]. Additionally, nanotechnology contributes to mitigating enteric methane (CH_4_) emissions through the manipulation of microbial populations and fermentation dynamics in the rumen. In parallel, the use of nano-sensors and nano-enabled diagnostics offers real-time monitoring of animal health, enabling more responsive and precise management strategies aligned with the principles of precision livestock farming [5].

Although this review centers on ruminants, it helps to briefly consider how nanotechnology behaves in monogastric animals, where digestion occurs in a single-chambered stomach and absorption takes place directly in the small intestine. This straightforward digestive pathway allows nano-formulated minerals and bioactive compounds to be absorbed more efficiently, often at lower doses than their conventional counterparts. Studies with zinc and selenium nanoparticles in pigs and poultry, for example, show improved mineral uptake, immune function, and growth performance [6]. Ruminants, on the other hand, depend on a multi-compartment stomach dominated by the rumen—a highly active microbial environment that can alter, degrade, or deactivate nano-supplements before they reach the intestine. This presents unique challenges but also opportunities. Nano-encapsulation can shield sensitive nutrients such as amino acids, fatty acids, or trace minerals, enabling them to bypass ruminal fermentation and reach the lower gut intact. When designed appropriately, nano-minerals can also support more efficient rumen fermentation, reduce methane output, and enhance nutrient use while limiting mineral excretion into the environment [7]. These contrasting physiological landscapes mean that a nanomaterial performing well in monogastrics may behave very differently in ruminants. Optimizing safety, dose, stability, and delivery therefore requires species-specific strategies rather than a one-size-fits-all approach.

Beyond nutrition, nanotechnological tools such as nano-tubes, nano-rods, and nano-fibers are being explored in areas like reproductive monitoring, disease detection, drug delivery, and vaccine development [1]. Their customizable properties allow targeted release, improved therapeutic efficacy, and enhanced immune responses [8].

Despite the growing promise, the application of nanotechnology in animal systems raises concerns regarding biosafety, environmental persistence, regulatory clarity, and economic feasibility [9]. The potential risks associated with NP accumulation in animal tissues, the environment, and the food chain remain under investigation [9]. This review provides a critical overview of nanotechnology’s role in ruminant nutrition and livestock production. It examines the advances in nano-formulated feed additives, delivery systems, and health-monitoring technologies, alongside the existing limitations and future research priorities. By bridging animal nutrition, biotechnology, and engineering disciplines, nanotechnology presents an exciting opportunity to reshape livestock farming into a more sustainable, efficient, and responsive sector capable of meeting global food security demands.

## 2. Methods

This review followed a systematic search approach rather than a traditional narrative review. The literature search was conducted systematically by a single researcher following PRISMA guidelines [10,11]. A predefined set of search terms, databases, and eligibility criteria guided the selection process. Only peer-reviewed journal articles published in English were considered. Databases searched included Scopus, ScienceDirect, Google Scholar, ResearchGate, Academia, and Wiley Online. Studies were included if they (i) were published within the search period; (ii) evaluated nanotechnology applications in ruminant species—cattle, sheep, goats, or buffalo; (iii) used in vitro, in situ, or in vivo experimental designs; and (iv) reported measurable outcomes related to rumen fermentation, nutrient utilization, animal performance, health, or safety. To ensure scientific rigor, only studies with clearly described methodologies, appropriate controls, and standard analytical procedures were retained. Studies that focused on monogastric species, non-nanotechnology additives, or lacked sufficient methodological detail were excluded.

The initial keyword search focused on nanotechnology applications in ruminant nutrition, including terms such as “nanoparticles,” “nano-minerals,” “nano-encapsulated bioactives,” “enzyme nanoparticles,” “nano-sensors,” “rumen fermentation,” “rumen microbiota,” “feed efficiency,” “nutrient utilization,” “animal performance,” “milk production,” “growth performance,” and “animal health.” Titles and abstracts were further screened for specific terms such as “nano-delivery systems,” “nano-packaging,” “chelation,” “bioavailability,” “nano-encapsulation,” and “nano-mineral supplementation.”

All retrieved articles were manually reviewed for relevance. Studies were excluded if they were duplicates, not aligned with the review objectives, or focused on non-nanotechnology feed additives. In addition to these criteria, each eligible study underwent a structured quality assessment to ensure reliability of the evidence, particularly for biological, physiological, or toxicological outcomes. Quality evaluation considered (i) clarity of experimental design; (ii) appropriate selection of control groups; (iii) replication and sample size; (iv) detailed reporting of nanomaterial characteristics (e.g., size, shape, dose, synthesis method); (v) validated analytical procedures; and (vi) statistical rigor. Studies lacking essential methodological information, those with unclear endpoints, or those using unstandardized toxicity testing protocols were excluded. For in vivo toxicological studies, only articles that described animal welfare considerations, ethical approvals, and standardized biomarker assays were retained.

Data was extracted according to the following key questions: (i) What type of nanomaterial or nano-formulation was applied, and how was it synthesized (physical, chemical, or biological/green methods)? (ii) Did the nanomaterial affect rumen microbial composition or fermentation parameters? (iii) Did it influence nutrient utilization, feed efficiency, or animal performance? (iv) Were there effects on animal health, immunity, or product quality? (v) What factors influenced the response, including dose, form, or duration of supplementation? (vi) Were there any adaptations of rumen microbiota to long-term nano-material exposure? (vii) Was safety, environmental, or regulatory considerations addressed?

This systematic approach ensured that the included studies met methodological standards, minimized bias, and provided a comprehensive assessment of the applications, mechanisms, and implications of nanotechnology in ruminant nutrition and production systems.

Unlike previous reviews that mainly focused on either nutritional outcomes or specific nano-material applications, this review integrates multiple dimensions of nanotechnology, including nano-material classification, physicochemical properties, synthesis methods (physical, chemical, and green biological approaches), and nanotechnology-related safety and environmental considerations. Building on this foundation, the present review extends beyond earlier work by incorporating recent findings published between 2022 and 2025, including advances in nano-enabled methane mitigation, nano-sensor–supported precision feeding, microencapsulated phytochemicals, and long-term safety evaluations of nano-minerals in ruminants. It also brings forward emerging applications in reproduction, immunity, and mycotoxin detoxification, creating a more comprehensive and forward-looking synthesis than previous publications. By combining mechanistic insights, practical applications, and regulatory perspectives, this review provides a holistic and up-to-date understanding of how nanotechnology can optimize rumen function, nutrient utilization, and animal performance, while identifying knowledge gaps and offering guidance for safe and effective implementation in ruminant production.

## 3. Synthesis and Preparation Methods

**Figure 1:**
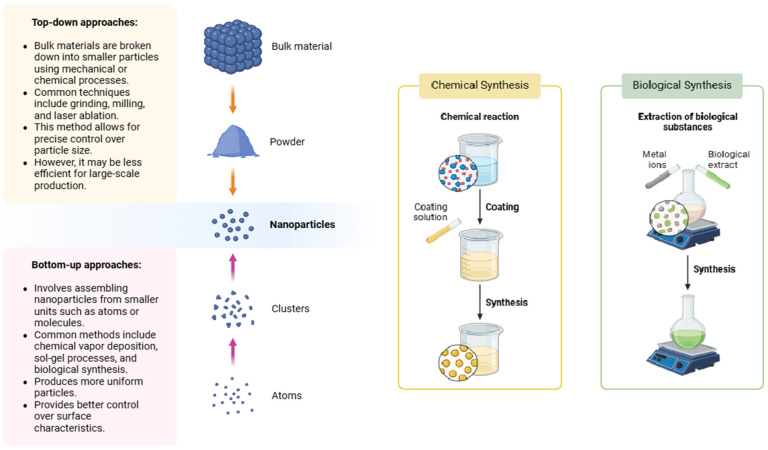
Methods of nanoparticle synthesis. Schematic representation of chemical and biological (green) synthesis approaches, highlighting top-down and bottom-up strategies.

Nanoparticles can be synthesized through various methods (Figure 1), each offering advantages in terms of scalability, cost, and environmental impact. Broadly, synthesis methods can be divided into top-down and bottom-up approaches [12].

### 3.1. Top-Down and Bottom-Up Approaches

In the top-down approach, bulk materials are broken down into smaller particles using mechanical or chemical processes. Techniques such as grinding, milling, and laser ablation are commonly employed. This approach allows for precise control over particle size but can be less efficient for large-scale production [12]. In contrast, the bottom-up approach involves assembling NPs from smaller units such as atoms or molecules [12]. Methods such as chemical vapor deposition, sol–gel processes, and biological synthesis are employed. This approach generally offers more uniform particles with better control over their surface characteristics.

### 3.2. Green Synthesis

An emerging trend in nanotechnology is the biological (green) synthesis of NPs, which uses plants, bacteria, and fungi as natural catalysts [2]. This environmentally friendly approach reduces the need for toxic chemicals, making it an attractive option for sustainable NPs production in agriculture [13]. The process utilizes plant extracts rich in bioactive compounds such as proteins, carbohydrates, polyphenols, alkaloids, terpenoids, and other phytochemicals, which serve as natural reducing and stabilizing agents for metal ions during NPs formation [2].

### 3.3. Industrial Preparation Techniques

A variety of NPs synthesis methods—such as physical, chemical, reactive precipitation, sol–gel, microemulsion, and supercritical chemical processing—have been extensively documented in the literature as effective approaches for tailoring NPs to specific applications in animal nutrition [14].

*Cross-linking Emulsion Method*: This technique is widely used for fabricating polymer-based NPs. It involves forming a water-in-oil emulsion, followed by chemical or ionic cross-linking to solidify the polymeric matrix, entrapping the active agents [15]. Cross-linking agents such as glutaraldehyde or calcium ions are frequently employed to stabilize the NPs, ensuring sustained release and improved gastrointestinal stability [14].

*Precipitation and Coacervation Methods*: These are classical bottom-up synthesis techniques. Precipitation relies on the supersaturation of a solution leading to nucleation and growth of NPs, often used for inorganic NPs like nano-Se or nano-Zn [14]. Coacervation refers to phase separation in polymer solutions, forming a dense, polymer-rich phase where NPs can be encapsulated, which is advantageous for encapsulating heat-sensitive bioactives [16].

*Spray-Drying Technology*: This is a well-established and scalable technique for producing dry NPs powders from liquid suspensions. It involves atomizing a liquid feed into a hot gas stream, where rapid solvent evaporation forms dry particles with controlled attributes [17]. It is particularly suitable for preparing feed-grade NPs formulations intended for oral delivery, such as microencapsulated essential oils, probiotics, or trace minerals.

## 4. Principles and Classification of Nanomaterials in Animal Nutrition

Nanotechnology, which involves the manipulation of matter at dimensions ranging from 1 to 100 nanometers [1,8], is revolutionizing animal nutrition, particularly for ruminants [2,3]. By harnessing the unique properties exhibited by materials at the nano-scale, nanotechnology is enhancing the effectiveness of nutrient delivery, improving the stability and absorption of supplements, and reducing the environmental impact of livestock production [2]. These innovations are especially beneficial in the complex digestive systems of ruminants, where nano-materials can interact with biological systems at a much more precise level than conventional methods [1,3].

The fundamental advantages of nanomaterials stem from their unique physical, chemical, and biological properties compared to larger particles, such as enhanced material strength, solubility, conductivity, optical properties, thermal behavior, and catalytic activity. Their larger surface-to-volume ratio and greater number of atoms at the surface contribute to these distinctive attributes [18]. The higher surface curvature and increased catalytic sites make NPs more reactive than their bulk counterparts, leading to different biological consequences compared to larger particles. This enhanced reactivity and high bioavailability allow for lower dosages while maintaining or even improving the effectiveness of the delivered compounds, thereby enhancing animal performance, reducing waste, lowering costs, and minimizing the environmental burden associated with livestock production.

### 4.1. Classification and Structural Comparisons of Nanomaterials

Nanomaterials—such as NPs, nano-emulsions, and nano-capsules—are gaining increasing attention in ruminant nutrition due to their distinctive physicochemical properties, including high surface area, enhanced reactivity, and improved solubility, which promote more efficient nutrient interactions and biological responses [2]. These materials are typically categorized into inorganic, organic, emulsified, and clay-based forms, each offering specific functional benefits.

#### 4.1.1. Metal-Based Nanoparticles (Inorganic)

Metal-based NPs such as zinc oxide (ZnO), selenium, copper (Cu), silver, and chromium have emerged as innovative tools in animal nutrition. Their nano-scale size—typically below 100 nm—grants them enhanced surface area, reactivity, and absorption efficiency, thereby improving mineral bioavailability, nutrient synergy, and retention in animal diets [2,19]. These attributes allow for lower dosages, reduced feed costs, minimized mineral excretion, and consequently, a lower environmental footprint [20]. Inorganic NPs, especially those formulated with trace minerals, are widely applied in feed additives, antimicrobial agents, water purification, and packaging to prolong shelf life and enhance feed hygiene [3].

#### 4.1.2. Nano-Capsules and Organic Nanoparticles

Nano-capsules are core–shell nano-structures with a hollow center capable of encapsulating a wide variety of substances. Their core functionality lies in protecting these substances from degradation and enabling their controlled, targeted release within the digestive tract. In animal nutrition, nano-capsules have primarily been employed to encapsulate feed additives, enhancing their delivery and efficacy. These improvements stem from the nano-capsules’ ability to shield the bioactive compounds from environmental stressors such as pH fluctuations, enzymatic degradation, light, and oxygen. Moreover, nano-encapsulation enhances the solubility and bioavailability of poorly water-soluble compounds—particularly lipophilic molecules [20].

Organic NPs, often composed of encapsulated proteins, lipids, or carbohydrates, improve nutrient bioavailability and can function as biosensors or antimicrobial elements in smart packaging [2]. Among the nano-materials used, chitosan—a natural polysaccharide derived from crustacean shells—has been the most frequently employed polymer for encapsulation in animal nutrition. Chitosan is biodegradable, biocompatible, non-toxic, and environmentally safe, making it highly suitable for feed applications. Chitosan nano-capsules effectively protect bioactive compounds and minerals through the rumen or gastric environment, allowing for their intact release and function in the small intestine [21].

#### 4.1.3. Nano-Emulsions

**Figure 2:**
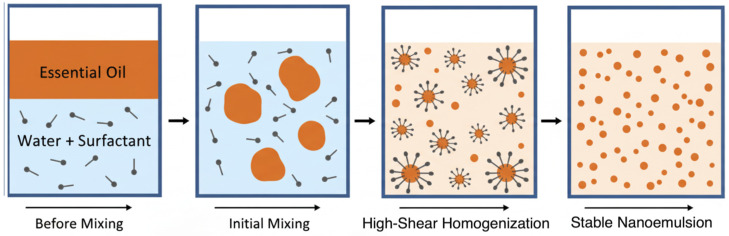
Illustration of the nano-emulsion formation process, showing how essential oils are dispersed into an aqueous phase and stabilized by surfactants during high-shear homogenization.

Nano-emulsions are colloidal dispersions characterized by droplet sizes typically ranging from 20 to 200 nm, with some systems featuring droplets smaller than 100 nm [22,23] (Figure 2). These systems consist of one liquid dispersed in another immiscible liquid and are stabilized by surfactants. Due to their extremely small droplet size, nano-emulsions exhibit unique physicochemical properties, including kinetic stability, optical transparency, a high surface area-to-volume ratio, and tunable rheological behavior [21]. These characteristics make nano-emulsions highly suitable for the encapsulation and delivery of lipophilic and bioactive compounds, such as essential oils, fatty acids, and fat-soluble vitamins, especially in animal nutrition. The application of nano-emulsions in ruminant diets enhances the bioavailability, stability, and efficacy of encapsulated compounds by improving gastrointestinal absorption and protecting sensitive ingredients from environmental degradation (e.g., oxidation, light, and pH) [2,24].

## 5. Applications of Nanotechnology in Ruminant Production

Nanotechnology is increasingly being applied in ruminant nutrition to address several challenges, such as improving the efficiency of nutrient utilization, enhancing feed safety, mitigating greenhouse gas emissions, and promoting animal health. Nano-materials’ unique properties, such as their high surface area and the ability to interact at the molecular level, make them particularly useful for modulating feed characteristics and animal responses. This section explores the primary applications of nanotechnology in ruminant feeding systems. Sources and applications of nano-feed additives are shown in Table 1.

### 5.1. Enhanced Nutrient Delivery and Bioavailability (Nano-Minerals)

Minerals are essential for various physiological functions in ruminants, including bone formation, immune competence, and reproductive performance [29]. However, conventional mineral supplements are often limited by low bioavailability and antagonistic interactions within the rumen, leading to suboptimal absorption [29]. Nanotechnology has emerged as a promising approach to address these limitations by enhancing the solubility, absorption, and overall bioavailability of minerals, particularly trace elements such as zinc, selenium, and copper [2].

Nano-minerals offer several advantages in ruminant nutrition. Their small size facilitates faster absorption compared to conventional mineral forms, thereby improving production efficiency and meeting metabolic mineral demands more effectively [24]. In addition, nano-minerals reduce intestinal mineral antagonism, minimize excretion, and lower environmental contamination. They also serve as potential alternatives to low-dose antibiotics, contributing to enhanced growth performance, reduced chemical residues, and the production of environmentally sustainable animal products [30]. Effects of some nano-minerals on ruminant performance are shown in Table 2.

*Zinc NPs (nano-Zn)*: Nano-Zn has been the focus of multiple studies, especially in poultry systems, due to their role in enhancing growth, immune response, antioxidant capacity, and meat quality. In ruminants, studies have shown that nano-Zn can enhance the concentrations of volatile fatty acids (VFAs), microbial crude protein synthesis, and the fermentation efficiency of OM in vitro [31]. Furthermore, it has been associated with an increase in milk production and a significant reduction in somatic cell count in lactating cows [32], as well as the elimination of damaged spermatozoa and improved semen quality in buffalo [33]. However, care must be taken, as high levels may induce toxicity, oxidative stress, or tissue damage [37].

*Selenium NPs (nano-Se)*: Nano-Se plays a vital role in redox balance and immune function. In ruminants, nano-Se has been associated with reduced greenhouse gas emissions by modulating rumen fermentation [35,38]. Specific research indicates that higher concentrations of nano-Se can enhance the in vitro digestibility of DM and decrease total gas and CH_4_ production [35]. In dairy cows, nano-Se supplementation has led to a linear increase in total VFA concentrations and quadratic improvements in the digestibility of DM, OM, CP, NDF, and ADF [36]. Additional benefits include protective effects against aflatoxin B1 and chromium-induced toxicity [39].

*Silver NPs (nano-Ag or Ag-NPs)*: Nano-Ag have been extensively studied for their strong antimicrobial properties. While primarily studied in poultry, their mechanism of action suggests potential as a topical or feed additive to manage bacterial load in ruminant environments, though excessive exposure may lead to tissue accumulation, neurotoxicity, and oxidative stress, indicating a need for regulated usage.

*Chromium NPs (nano-Cr)*: Chromium tripicolinate NPs have shown efficacy in improving feed intake, nutrient digestibility, antioxidant status, and immune parameters under stress conditions, especially heat stress [40]. These effects are partly attributed to chromium’s role in carbohydrate and lipid metabolism, which supports homeostasis during physiological stress.

### 5.2. Nano-Encapsulation for Controlled Release of Feed Additives

Encapsulation involves enclosing bioactive substances, such as enzymes, probiotics, essential oils, and vitamins, within a protective layer to enhance their stability, controlled release, and bioavailability [2]. Nanotechnology facilitates the development of nano-sized encapsulating carriers that offer significant advantages over conventional delivery methods. Specific applications of nano-encapsulation include:

*Protection of Bioactive Compounds*: The use of nano-capsules is crucial for transporting and releasing nutrients in a controlled manner. For example, nano-chelates around 50 nm in size have been found to efficiently deliver nutrients without altering the feed’s color or taste, ensuring optimal nutrient uptake without compromising feed quality [41]. The application of naturally occurring bioactive compounds as alternatives to antibiotic growth promoters often suffers from limited water solubility and instability; nano-capsulation offers a promising strategy to overcome these challenges, preserving their functional properties and enhancing their application in animal feed.

*Probiotic and Prebiotic Delivery*: Nano-encapsulation of probiotics, such as *Lactobacillus* and *Bifidobacterium*, can protect these microorganisms from degradation in the acidic environment of the rumen, ensuring their viability and effectiveness in the intestines [25]. Additionally, encapsulating prebiotics like oligosaccharides can improve their stability and promote the growth of beneficial ruminal microbes [26].

*Phytogenic Delivery*: Phytogenic compounds, such as essential oils derived from plants like oregano, garlic, and cinnamon, are recognized for their antimicrobial and antioxidant properties [42,43,44]. Nano-encapsulation of these compounds enhances their solubility and stability in ruminant diets, allowing for better integration into the rumen [45]. This integration helps modulate microbial activity, improve fermentation efficiency, and reduce the occurrence of subclinical infections [2,45].

### 5.3. Mode of Action and Antimicrobial Properties

The functional mechanisms of NPs in ruminant nutrition are principally centered on their capacity to transport various bioactive compounds under diverse environmental conditions. Their nano-scale size allows them to pass through capillary walls and epithelial linings, thereby enabling targeted delivery of nutrients, enzymes, and therapeutic agents. These characteristics enhance biological interactions, prolong compound residence time, reduce intestinal clearance, and facilitate tissue penetration [2].

**Figure 3:**
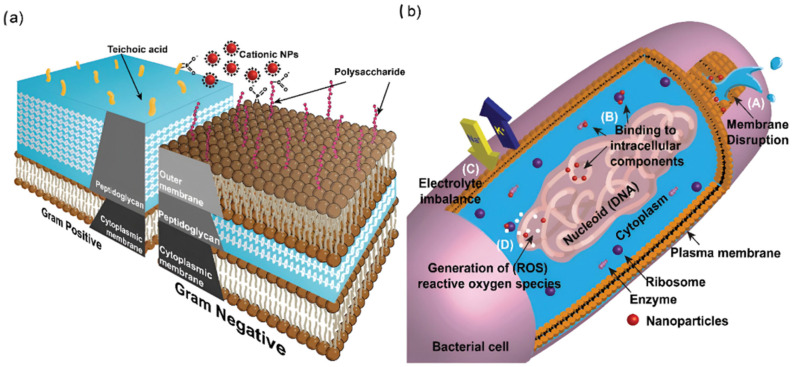
Schematic illustration of the antibacterial effects of nanoparticles. Panel (**a**) shows the cell wall structures of Gram-positive and Gram-negative bacteria, while panel (**b**) summarizes the main antibacterial mechanisms of nanoparticles: (**A**) disruption of the cell membrane causing cytoplasmic leakage, (**B**) binding to and disruption of cellular components, (**C**) interference with electron transport leading to electrolyte imbalance, and (**D**) induction of ROS. Reproduced from Gupta et al. [54] with permission from the Royal Society of Chemistry.

*Antimicrobial Mechanism*: NPs, particularly metal and metal-oxide types, exhibit potent antimicrobial activity through multifaceted mechanisms (Figure 3). These include disrupting bacterial membranes, inhibiting enzymes, interfering with gene expression, and generating reactive oxygen species (ROS), which cause oxidative stress and cellular damage [46]. Their electrostatic interaction with negatively charged bacterial walls enhances biosorption and bactericidal action. Gram-negative bacteria are generally more susceptible to NP penetration than Gram-positive types due to structural differences [47]. Additionally, NPs can serve as antibiotic carriers, improving drug efficacy while reducing dosage requirements [46].

Importantly, there is emerging rumen-specific data that supports and refines these antimicrobial mechanisms. For instance, a recent in vitro study found that nano-ZnO significantly reduces microbial populations in rumen fluid and decreases CH_4_ and carbon dioxide emissions in a dose-dependent manner, suggesting that nano-ZnO may directly affect ruminal fermentative bacteria [48]. In vivo, dietary supplementation of ZnO nanoparticles in dairy goats altered rumen microbiota composition—increasing beneficial groups such as *Prevotella* while reducing potentially harmful taxa [49].

These rumen-specific results imply that nanoparticles may modulate microbial communities in ways beyond broad-spectrum killing—potentially reshaping fermentation profiles, influencing microbial diversity, and shifting metabolic activity. Such effects may result not only from bactericidal activity but also from subtler microbial modulation. Therefore, it is justified to expand the antimicrobial mechanisms discussion in this paper to reflect both classical modes (membrane disruption, ROS, ion release) and rumen-targeted effects.

Recent primary studies in ruminants provide direct evidence that nanoparticles can modulate the rumen microbiota. For example, dietary ZnO-NPs in lambs reduced total bacterial populations and altered fermentation profiles and rumen histology [50]. In dairy goats, ZnO-NP supplementation increased milk yield and shifted microbial composition, notably boosting *Prevotella* and *Rikenellaceae* groups [49]. Beyond broad-spectrum effects, engineered functional nanoparticles offer targeted interventions: biological PHB nanoparticles loaded with a lytic enzyme significantly reduced methane-producing archaea in rumen models [51]. Furthermore, plant-mediated Ag-NPs (from beetroot extract) administered to lambs influenced rumen pH, total bacteria and protozoa, and VFA concentrations [52]. Finally, lipid-based nanocarriers (e.g., solid lipid nanoparticles) can survive ruminal digestion and protect nutrients like lysine from microbial degradation, improving post-ruminal delivery [53].

These studies suggest that, in the rumen, nanoparticles can act not only as antimicrobials but also as modulators of fermentation and community structure. This nuanced mechanistic understanding reinforces their potential in precision nutrition strategies for ruminants.

*Specific Metal NP Mechanisms*: Among specific NPs, nano-Ag demonstrate broad-spectrum antimicrobial action by releasing silver ions that interfere with respiration and protein synthesis, while also enhancing antibiotic activity via ROS generation [55]. Gold NPs (Au-NPs), with preferential action against Gram-negative bacteria, can operate through ROS-independent mechanisms, potentially lowering mammalian toxicity [56]. Titanium dioxide and ZnO rely on photocatalytic ROS production, disrupting bacterial membranes, deoxyribonucleic acid (DNA), and metabolic processes [57]. Other NPs like iron oxide, platinum, copper, selenium, and magnesium oxide (MgO) also exhibit bactericidal effects through mechanisms such as ion release, ROS generation, and interference with vital cellular functions and metabolic pathways [46].

## 6. Animal Performance

Feed efficiency is a critical factor in the cost-effectiveness of livestock production. Nanotechnology offers several ways to improve the efficiency of feed utilization in ruminants, leading to reduced feed costs and improved growth and milk production.

### 6.1. Mycotoxin and Aflatoxin Mitigation Through Nanotechnology

Mycotoxins, particularly aflatoxins, are toxic secondary metabolites produced by filamentous fungi, primarily species of *Aspergillus*, *Penicillium*, and *Fusarium* [58]. These contaminants are prevalent in a wide range of feedstuffs, including cereals, oilseeds, and forages, and pose serious risks to animal health, performance, and the safety of animal-derived food products [58]. Among these, aflatoxin B1 is the most potent and widely studied, recognized as a Group 1 carcinogen by the International Agency for Research on Cancer (IARC) due to its hepatotoxic and immunosuppressive effects. Traditional mycotoxin mitigation strategies—such as physical separation, chemical detoxification, and the use of adsorbents like bentonite clays, activated carbon, and yeast cell wall extracts—offer limited efficacy, often lack specificity, and may inadvertently bind essential nutrients, thereby reducing feed efficiency and in-creasing economic losses. Nanotechnology provides new opportunities by introducing nano-structured binders, nano-enabled detoxification systems, and nano-based sensing platforms that offer higher specificity and functional versatility than conventional approaches.

Nanotechnology enables the design and application of nano-materials with tailored physicochemical properties, which facilitate mycotoxin control across the entire production chain, from detection to binding and detoxification. The initial and most critical step is the rapid and sensitive detection of contaminants, which is achieved through the development of nano-sensors. These sensors—based on quantum dots, Au-NPs, or metal–organic frameworks—enable highly sensitive, on-site detection of aflatoxins in feed matrices, facilitating timely intervention and reducing the risk of exposure [59].

Following detection NPs can act in two distinct functional roles:

(1) Nanoparticles as binders (adsorbents): Nano-silica, nano-clays (e.g., montmorillonite, halloysite), and carbon-based nanomaterials (e.g., graphene oxide, carbon nanotubes) primarily function as high-affinity binders. Their large surface area, tunable charge, and surfaces that can be functionalized, allow them to selectively adsorb aflatoxins within the gastrointestinal tract, preventing absorption into systemic circulation. Unlike conventional binders, these nano-adsorbents can be engineered to minimize interactions with nutrients, reducing the risk of unintended nutrient sequestration [58].

(2) Nanoparticles as detoxifiers or carriers of detoxifying agents: A second category includes NPs that actively degrade or bio transform aflatoxins. Nano-encapsulation systems—such as liposomes, chitosan NPs, and polymeric nanospheres—can deliver enzymes (e.g., laccases), microbial strains, or bioactive metabolites that catalyze aflatoxin breakdown. This approach represents “true detoxification,” where the toxin is chemically converted into less harmful metabolites rather than merely bound. For example, bioactive secondary metabolites derived from *Bacillus subtilis*, when encapsulated in nano-emulsions or alginate-based systems, have shown superior degradation of aflatoxins and reduced hepatic oxidative stress in poultry and ruminants [60].

In addition to binding and detoxification, certain functional NPs (e.g., nano-Ag) may mitigate aflatoxin-induced oxidative damage and immunosuppression, thereby improving performance measures such as body weight gain, feed intake, feed conversion ratio, serum biochemical indices, and hepatic tissue integrity [61]. These effects represent mitigation of toxicity rather than direct detoxification of the aflatoxin molecule. To address concerns about digestibility versus binding activity, it is important to distinguish that nutrient-digestibility-enhancing NPs (e.g., nano-minerals or nano-enzymes) differ fundamentally in structure and function from nano-adsorbents used to bind aflatoxins. Therefore, the same NP is not simultaneously expected to enhance digestibility and bind toxins. Instead, targeted formulations allow each NP class to fulfill its intended role without undesirable cross-interactions.

### 6.2. Nano-Sensors and Precision Feeding

Quantifying the digestive and fermentative processes within the rumen has long been a focus of ruminant nutrition research; however, existing methodologies often lack the temporal and spatial resolution necessary to fully capture the dynamic nature of this complex ecosystem. Nano-sensors, in particular, offer transformative potential for non-invasive, real-time monitoring of rumen parameters and broader physiological indicators [5]. These sensors can be embedded in boluses, fistulated devices, or wearable tools to continuously monitor rumen pH, temperature, microbial composition, and feed intake. Such integration facilitates precision feeding, whereby diets are dynamically adjusted based on individual animal needs. Table 3 provides a summary of the current status and applications of nanoparticle-based sensors in ruminant production.

Real-time, in situ monitoring using implantable microsensor technologies presents a promising approach to overcoming these limitations, enabling the continuous assessment of key chemical and physical parameters such as pH, temperature, VFAs, and histamine concentrations [5]. These microsensors not only facilitate deeper understanding of ruminal metabolism but also hold potential for advancing precision livestock farming, particularly when integrated with robust wireless data transmission and analytics platforms. For example, nano-sensors can detect early deviations in feed intake or core body temperature, allowing for timely interventions in cases of metabolic disorder or infection [5]. In the case of VFAs, biosensors employing microbial or enzymatic biocatalysts show promise but often fail to operate effectively within biologically relevant concentration ranges. Moreover, selectivity remains a significant hurdle, as few sensors can reliably distinguish among different VFAs [62].

Furthermore, monitoring the rumen microbiome using nano-sensors enables the detection of beneficial and pathogenic microbial populations [63], offering opportunities to fine-tune dietary inputs to promote microbial balance and improve fermentation efficiency. This, in turn, can enhance nutrient utilization and mitigate CH_4_ emissions. When combined with automated feeding systems and predictive data analytics, nano-sensors support the development of precision feeding systems capable of optimizing animal health, productivity, and environmental sustainability.

Beyond sensor development, a critical but under-addressed challenge lies in the interpretation and application of sensor-generated data. The biological meaning of data streams—such as those related to pH, temperature, and feed intake—is difficult to contextualize without reference standards or high-density sampling alternatives [5]. For instance, while fluctuations in temperature may signal estrus or illness, similar patterns may result from variations in feed or water intake, environmental conditions, or stress, complicating data-driven decision-making. Thus, multimodal sensing—combining signals from various physiological indicators—may be required to provide sufficient contextual information for actionable insights. However, sensor development must go beyond technical performance metrics to include data usability, farm-level applicability, and transparent analytical frameworks.

### 6.3. Nutrient Digestion and Absorption

Nano-based carriers have emerged as innovative tools in animal nutrition, offering controlled and slow-release delivery systems for nutrients, thereby enhancing their bioavailability and reducing nutrient losses in ruminant animals. These carriers, including liposomes, polymeric NPs, nano-emulsions, and nano-clays, can encapsulate vitamins, minerals, amino acids, and fatty acids, protecting them from degradation in the rumen and ensuring their targeted release in the intestine. Such controlled-release systems also reduce the frequency of nutrient supplementation, improving feed efficiency and lowering production costs [21].

NPs administered orally must first negotiate the mucus layer that coats the intestinal epithelium, which serves as a dynamic and selective barrier. Goblet-cell–derived mucins form a mesh-like network with pore sizes typically ranging from ~100 to 200 nm, and negatively charged glycosylated groups that can trap particles via electrostatic or hydrophobic interactions [64]. The fate of NPs in this mucosal environment depends heavily on their physicochemical properties—notably size, surface charge, hydrophilicity, and surface coating [65]. For instance, NPs with dense, neutral hydrophilic coatings (such as low-molecular-weight polyethylene glycol) can diffuse more readily through mucus by minimizing adhesive interactions [66]. In contrast, cationic or hydrophobic particles may become strongly trapped via electrostatic binding to mucins, reducing their mobility and potentially limiting their uptake [67]. Once NPs penetrate or adhere to the mucus, they can engage with the epithelium via several routes: (i) transcytosis through enterocytes or M cells, (ii) paracellular transport via tight junctions, or (iii) receptor-mediated endocytosis if they are functionalized with targeting ligands [68]. Importantly, mucus-adherent particles that cannot penetrate often get cleared as the mucus layer is continuously renewed, a process that can limit long-term absorption [69]. In ruminant systems, these interactions may be further complicated by the unique gut physiology (e.g., the rumen, high microbial load) and the feed matrix. Thus, understanding and engineering NP surface properties to balance mucus penetration and epithelial uptake is critical to designing effective nano-supplements for ruminants.

Nanotechnology presents a transformative opportunity in ruminant nutrition by enhancing the digestion, absorption, and overall utilization of nutrients [2]. In conventional feeding systems, many essential nutrients—such as vitamins, amino acids, enzymes, and phytochemicals—are prone to degradation in the harsh environment of the rumen, significantly reducing their bioavailability. To address this challenge, nanotechnology offers advanced delivery systems like nano-encapsulation, which protect these compounds and allow for their controlled release at targeted sites in the gastrointestinal tract, particularly the intestine where absorption is most efficient [2,21].

Natural and synthetic NPs, including protein-based carriers like casein and lipid-based systems, serve as stable transport vehicles for bioactive compounds. Casein micelles, for example, have been shown to effectively carry nutrients such as calcium, vitamin D, and proteins, supporting efficient absorption and biological processes such as maternal nutrient transfer [70]. The nano-scale size of these particles enables them to penetrate the intestinal mucosa more easily than larger particles. This enhances interaction with the intestinal lining and promotes faster, more efficient nutrient uptake [71].

Nanotechnology also improves the stability and solubility of poorly bioavailable compounds. Nutrients with low solubility, like certain fat-soluble vitamins and trace minerals, can be reformulated into water-dispersible nano-forms, significantly increasing their absorption and efficacy [72]. This increased efficiency reduces the need for high-dose supplementation, lowering feed costs and minimizing environmental waste. Furthermore, nano-carriers can bypass physiological barriers and deliver nutrients directly to target tissues, optimizing their use and improving animal productivity [4].

Beyond nutrient delivery, nanotechnology plays a critical role in modulating the ruminal microflora—an essential component of the ruminant digestive system [21,73]. The rumen hosts a diverse microbial community, including bacteria, protozoa, fungi, and archaea, responsible for breaking down fibrous plant material and converting it into VFAs and microbial protein. Nano-particles can selectively influence this microbial ecosystem. For instance, metal-based NPs such as ZnO or silver have antimicrobial properties that may suppress harmful or less beneficial microbes while promoting fiber-degrading bacteria [27]. Additionally, nano-encapsulated additives like essential oils, enzymes, or probiotics can be protected from ruminal degradation, enhancing their impact on microbial balance and activity [73].

Such modulation can improve feed digestibility, reduce CH_4_ emissions by targeting methanogenic archaea, and enhance nutrient utilization. For example, Muslykhah et al. [73] demonstrated that microencapsulated duckweed extract altered rumen microbial populations in vitro by significantly reducing the abundance of *Ruminococcus albus*, *Ruminococcus flavefaciens*, and *Methanobacteriales*, compared to both unencapsulated duckweed powder and a control. These changes suggest that nano-encapsulation may enable precise targeting of microbial groups involved in fiber breakdown and hydrogen utilization, contributing to a more efficient and environmentally sustainable digestive process.

### 6.4. Ruminal Fermentation and Methane Production

Nanotechnology has emerged as a powerful tool in animal nutrition, particularly for enhancing ruminal fermentation efficiency and mitigating CH_4_ emissions. The unique physicochemical properties of NPs—especially their high surface-area-to-volume ratio and nano-scale size—enable them to interact closely with microbial cells and fermentation substrates, thus influencing rumen microbial ecology and fermentation pathways [38,73]. Nano zeolite increased ruminal pH and stimulated microbial activity, as indicated by a higher total protozoal count and a greater abundance of *Diplodinium* spp. ciliates [74].

These additives exert selective antimicrobial activity, suppressing harmful or CH_4_ -producing microbes while preserving or stimulating beneficial microbial communities [74]. For example, silver and ZnO-NPs can inhibit methanogens and proteolytic bacteria, thereby reducing ruminal NH_3_ concentrations and CH_4_ output, which in turn enhances nitrogen retention and feed efficiency [75]. Additionally, nano-encapsulated essential oils and phytochemicals promote favorable shifts in VFA profiles—particularly increasing propionate and decreasing acetate and CH_4_ production—supporting better energy metabolism in ruminants [31].

Nano-minerals such as ZnO, copper, and selenium have shown the ability to alter the abundance and activity of key microbial groups in the rumen. For instance, nano-Zn has been associated with increased populations of *Ruminococcus albus* and *Fibrobacter succinogenes*, leading to enhanced fiber degradation and elevated acetate production [50]. Moreover, nano-Zn supplementation has demonstrated positive effects on enzyme activity, DM digestibility, and NH_3_-N reduction across both short- and long-term studies in lambs, underscoring its influence on microbial functionality and nutrient utilization [50]. A recent study [50] investigated the impact of zinc nanoparticle supplementation on ruminal fermentation, microbial dynamics, and tissue histopathology in lambs using in vitro assays, a 28-day short-term trial, and a 70-day long-term feeding experiment. In the long-term trial, lambs received either a basal diet or diets supplemented with ZnO-NPs at 40 or 80 mg Zn/kg, or conventional ZnO at 80 mg Zn/kg. In the short-term trial, ZnP-NPs significantly reduced total bacterial populations without affecting protozoal counts. During long-term feeding, the highest ZnO-NP dose produced the lowest ruminal ammonia-N concentrations, and all Zn-NP treatments increased fibrolytic enzyme activities, including carboxymethyl cellulase and xylanase.

Beyond nutrient digestion, NPs can shift the production profile of VFAs—the primary energy source for ruminants. El-Nile et al. [74] reported that nano zeolite supplementation significantly increased the total concentration of VFAs, particularly butyric acid, while having no effect on the relative proportions of the other individual VFAs. Wei et al. [36] stated that nano-Se supplementation at doses of 0, 0.1, 0.3, and 0.5 mg/kg of DM in Holstein dairy cows resulted in a linear increase in total VFA concentrations and the molar ratios of propionate and butyrate. It also decreased rumen pH, NH_3_-N concentration, and the acetate to propionate ratio. The 0.3 mg/kg treatment group showed higher propionate levels. Additionally, nano-Se supplementation quadratically improved the digestibility of DM, OM, CP, NDF, and ADF, and linearly increased the absorption of total Se.

Nano-encapsulated plant extracts and essential oils have been shown to increase propionate at the expense of acetate, improving energy efficiency and reducing hydrogen availability needed by methanogens [73]. This modulation of fermentation pathways directly contributes to reduced CH_4_ synthesis in the rumen. Muslykhah et al. [73] found that supplementation with microencapsulated duckweed extract influenced VFA profiles during in vitro fermentation. Specifically, it increased the concentration of propionic acid while reducing acetic acid levels, leading to a shift in the acetate-to-propionate ratio. Additionally, the supplementation affected butyric acid concentrations, with notable variations depending on the level and form of duckweed used.

One of the most promising aspects of nanotechnology in ruminant nutrition is its potential to significantly curb enteric CH_4_ emissions. Methane is primarily produced in the rumen by methanogenic archaea during microbial fermentation, representing a major source of anthropogenic greenhouse gas emissions [76]. Traditional additives like tannins and essential oils do inhibit methanogenesis, but their instability and low ruminal bioavailability often limit effectiveness [77,78]. Nano-encapsulation techniques overcome these limitations by enhancing the stability, solubility, and controlled release of bioactive compounds. For example, nano-encapsulated tannins have demonstrated superior efficacy in suppressing methanogen activity [43,73]. Muslykhah et al. [73] reported that supplementation with microencapsulated duckweed extract at levels of 2%, 4%, and 6% significantly reduced CH_4_ production in an in vitro fermentation study compared to both the control and duckweed powder treatments. The reduction was most notable with increasing supplementation levels, highlighting the potential of the encapsulated form to modulate fermentation processes associated with CH_4_ output.

Functional NPs, including metal oxides like MgO, ZnO, and Cu-NPs, offer additional avenues for CH_4_ mitigation. MgO has been found to promote microbial biomass synthesis and shift fermentation toward increased propionate, resulting in reduced CH_4_ output [79]. ZnO-NPs, beyond improving bacterial proliferation, have been linked to reduced gas production and better energy use efficiency [31]. Similarly, Cu-NPs have supported the growth of beneficial microbes and enhanced the concentration of polyunsaturated fatty acids like omega-3 and omega-6, while also decreasing CH_4_ emissions at appropriate dosages [80].

Carbon-based nano-materials and Ag-NPs have also demonstrated selective inhibition of methanogens under anaerobic conditions, suggesting a broad applicability of nanotechnology in modulating ruminal microbial populations and fermentation dynamics [81].

Furthermore, nanotechnology contributes to CH_4_ mitigation indirectly by improving nutrient digestibility and reducing feed wastage. Better feed efficiency means less undigested material for microbial fermentation, thereby reducing the substrate available for methanogenesis. This dual role of improving animal performance and lowering emissions highlights the sustainability potential of nano-materials in livestock production systems.

Taken together, these findings underscore the multifunctional potential of nanotechnology in ruminant nutrition—not only in enhancing fermentation efficiency and nutrient utilization but also in offering a potent strategy for reducing CH_4_ emissions. By targeting microbial metabolism, improving additive delivery, and reshaping fermentation profiles, nano-materials represent a forward-looking approach for sustainable animal agriculture [38,76].

### 6.5. Lactation Performance

In the context of animal performance, NPs have been shown to improve the bioavailability of nutrients, boosting growth rates, milk production and improving overall animal health. Nanotechnology offers transformative applications in dairy production by enhancing milk safety, therapeutic value, and nutritional quality. A primary contribution is the rapid and sensitive detection of foodborne pathogens and toxins. Nano-composites incorporating anti-*Staphylococcus aureus* antibodies, gold, and magnetic NPs enable detection of bacterial contamination in milk within 40 min [82]. Similarly, polyclonal antibody-based Au-NPs systems can detect aflatoxin M1 in under 10 min using immunochromatographic strips, providing fast and reliable toxin screening. These innovations significantly improve milk safety assurance [37].

In terms of therapeutic enhancement, nanotechnology enables more efficient treatment strategies for dairy cattle. Solid lipid NPs, such as those made from hydrogenated castor oil, have been used to extend the half-life of drugs like tilmicosin, allowing for sustained release, reduced dosing frequency, and minimized milk withdrawal periods in mastitis management [5]. Additionally, ZnO-NPs exhibit notable antimicrobial properties. Their application has led to reduced somatic cell counts and improved milk yield in subclinical mastitis cases [32,34]. ZnO-NPs show broad-spectrum antibacterial activity against *Staphylococcus aureus*, *Escherichia coli*, *Streptococcus* spp., and *Klebsiella pneumoniae*, attributed to their small size, high surface reactivity, and the toxic effect of released Zn ions on bacterial structures [31].

Furthermore, nanotechnology can enhance milk’s nutritional value by addressing the limitations of conventional calcium fortification. Furthermore, nanotechnology can enhance milk’s nutritional value by addressing the limitations of conventional calcium fortification. Traditional calcium additives often exhibit low solubility, leading to sedimentation, a chalky mouthfeel, and reduced bioavailability [83]. Fortification with oyster shell nano-powder overcomes these challenges due to its nano-scale size, which improves solubility and dispersibility, prevents sedimentation, and preserves the sensory and physicochemical properties of milk during storage [84]. The increased surface area of NPs also promotes superior calcium absorption and bioavailability compared to micro-sized counterparts [27]. While conventional ingredients can achieve basic fortification goals, the nano-formulation offers additional functional advantages that enhance efficacy and product quality [27]. In an experiment, Rajendran et al. [32] stated that dietary supplementation with nano ZnO in dairy animals has been shown to significantly enhance milk yield, bolster immunological function, and mitigate subclinical mastitis, as indicated by a marked reduction in somatic cell count values. Beyond health and nutrition, ZnO-NPs have also shown promise in modulating rumen fermentation. Inclusion at 100–200 mg/kg in vitro improved microbial protein synthesis, energy efficiency, and VFAs production, while reducing NH_3_-N and the acetate-to-propionate ratio, thus enhancing fermentation dynamics [31]. Recent work showed that supplementing mid-lactation Guanzhong goats with 30 mg/kg DM of ZnO-NPs increases both milk yield and milk fat content, while also enriching ruminal microbial richness and diversity. Notably, the rise in beneficial bacterial taxa was positively associated with metabolites involved in enhanced energy and nucleotide metabolism [49]. El-Nile et al. [74] found that supplementing dairy goats with nano zeolite significantly increased milk yield without affecting feed intake, pointing to better feed efficiency. While both natural and nano forms of zeolite reduced milk fat, protein, lactose, solids-not-fat, and somatic cell count, the nano form had unique effects on milk fat composition. It lowered eicosanoic acid levels and raised myristoleic acid, though it did not significantly impact total saturated, odd and branched-chain, or polyunsaturated fatty acids. These results suggest that nano zeolite may not only boost milk production but also enhance certain nutritional aspects of milk quality.

### 6.6. Immunity of Animals

Beyond nutrition, nanotechnology is also transforming animal production and veterinary practices. It has revolutionized disease detection, treatment, vaccine development, and drug administration [6]. Additionally, NPs are improving the management of nutrition and reproductive issues in ruminants, enhancing digestion, metabolism, and microbiota balance, which are critical for the overall health and productivity of animals [6].

The gut-associated lymphoid tissue within the gastrointestinal tract (GIT) acts as a crucial defense mechanism, serving as a barrier to protect the body from harmful substances and pathogens [85]. The interaction between NPs and the GIT is influenced by a range of biological and physicochemical factors. The biological effects of NPs are shaped by their size, surface area, number, aggregation state, charge, and surface coatings, all of which play a role in how they are absorbed and how they elicit biological responses [86].

In toxicological studies, the characterization of nano-materials is essential for understanding their potential effects on health. Minimum requirements for NPs characterization include key parameters such as particle size, surface area, particle count, degree of aggregation or agglomeration, surface charge, and the presence of surface coatings [86]. The production of ROS (oxidants) and the rate at which NPs are broken down in the body also influence their biological effects, further complicating the interpretation of results [87].

In vivo experiments often introduce significant variability due to factors such as species differences, strains, diet, housing conditions, dosage timing, circadian rhythms, and the composition of the endogenous microbiome. The careful reporting of these factors can enhance transparency and help reconcile inconsistent results between studies, facilitating better comparisons and conclusions [88]. Standardization of experimental parameters, such as through the Metabolomics Standards Initiative and ARRIVE guidelines, aims to bring consistency to particle toxicity research, improving the quality of the findings.

### 6.7. Blood Parameters

Nano-feed additives have demonstrated considerable potential in modulating blood biochemical parameters in ruminant animals, thereby contributing to improved metabolic health and physiological stability [24]. Due to their enhanced bioavailability and controlled-release characteristics, nano-formulated nutrients and additives can exert more pronounced systemic effects than their conventional counterparts. Several studies have reported that nano additives, such as nano minerals (e.g., zinc, selenium, and calcium), nano zeolites, and nano-encapsulated phytochemicals, can positively influence key blood metabolites [27,74]. These effects include increased serum calcium and total protein concentrations, improved antioxidant enzyme activity (e.g., superoxide dismutase and glutathione peroxidase), and reduced levels of liver enzymes such as aspartate aminotransferase and alanine aminotransferase, indicating reduced hepatic stress [74]. Additionally, reductions in blood cholesterol, triglycerides, and total lipids have been observed, suggesting improved lipid metabolism and energy balance in animals supplemented with nano-based additives. Such improvements are particularly beneficial during physiologically demanding periods, such as late gestation and early lactation, when metabolic disorders are most likely to occur.

### 6.8. Reproduction

Nanotechnology presents a promising frontier for enhancing reproductive efficiency in livestock by refining artificial insemination practices and addressing broader reproductive management challenges. As a cornerstone of modern livestock production, artificial insemination improves genetic diversity and facilitates trait selection [89]. The integration of nanotechnology into reproductive strategies can optimize outcomes through advancements in semen quality, gamete imaging, cryopreservation, gene transfer, nutrient delivery, and disease control [6,38]. These innovations not only address current limitations but also contribute to increased productivity and the sustainability of livestock systems. Nonetheless, further research is essential to ensure these technologies can be safely, effectively, and economically implemented in practical on-farm settings.

Nano-purification of semen represents another key application, where magnetic NPs coated with antibodies or lectins selectively remove damaged spermatozoa, enhancing the quality of insemination [33]. Odhiambo et al. [90] reported that offspring produced using semen nano-purified with peanut agglutinin from *Arachis hypogaea*- and ubiquitin-binding techniques appeared completely normal. Moreover, heifers born during the first year of this artificial insemination field trial exhibited fertility comparable to those sired by bulls using non-purified semen. This technique may also be refined to target specific sperm biomarkers predictive of higher fertility, further enhancing reproductive success [33].

Cryopreservation technologies benefit from nano-enabled semen extenders, which can replace traditional antibiotics—often detrimental to sperm motility—with antimicrobial and nutrient-delivering NPs [1]. Natural substances like honey, tomato juice, and sugarcane juice, known to improve sperm viability during storage, can be incorporated more effectively using NPs carriers [1]. These strategies offer enhanced protection against oxidative and microbial damage during freezing and thawing, thereby improving post-thaw sperm quality. Since sperm can be transported internationally over several days, utilizing extenders with greater preservation capacity during freeze–thaw cycles is crucial—particularly if the functional groups of each product are delivered via NPs, which could otherwise affect sperm quality [91].

Further advancements in reproductive biotechnology may be achieved through the integration of NPs into molecular biology techniques. One promising approach is sperm-mediated gene transfer, where mesoporous silica NPs are used to deliver nucleic acids and proteins to spermatozoa without impairing their function or quality [92]. These NPs form strong associations with sperm in vitro and offer potential for targeted genetic modification and the propagation of desirable traits in offspring [92].

Additionally, polymeric NPs such as poly(2-dimethylamino)ethyl methacrylate (PDMAEMA), chitosan, and polyethylenimine have shown advantages over traditional viral transfection methods, provided they are used at low concentrations [93]. The molecular weight of these nano-polymers significantly affects both transfection efficiency and toxicity, with 60 kDa identified as the optimal weight for PDMAEMA-mediated transfection [93].

However, not all NPs are benign; some, like ZnO and titanium dioxide, have demonstrated spermatotoxic effects, including reduced sperm viability and DNA fragmentation in a dose- and time-dependent manner [94]. This highlights the need for careful NPs selection and comprehensive safety assessments to avoid adverse impacts on fertility.

## 7. Side Effects and Safety Validation Framework for Nanomaterials in Ruminant Production

Nano-particles have the potential to increase bioavailability and enhance digestive efficiency and growth performance; however, they may also pose risks such as inflammatory gastrointestinal diseases, altered nutrient absorption, and stability issues during heating or storage. One of the major challenges in assessing the toxicity of NPs is linking experimental results to real-world human health risks. This is particularly difficult due to the lack of precise environmental data and the inherent variability in how NPs interact in different biological systems. Additionally, extrapolating results from in vivo studies, which often use higher, shorter-term doses, to predict the effects of chronic, lower-dose exposure is complex and uncertain [2].

However, a review by Yip et al. [95] suggests that, in general, NPs appear to have low toxicity in in vivo tests, with no notable side effects observed at lower doses. For example, nano-Ag were tolerated at levels lower than 125 mg/kg without adverse effects [96], and up to 5000 mg/kg of TiO_2_ NPs were reported to be tolerated without significant negative effects [97]. Despite these findings, in vitro studies have shown cytotoxicity and increased membrane permeability, while in vivo studies generally indicate no significant effects except at high doses [98]. These mixed findings highlight the need for further research to establish clearer safety thresholds, particularly in relation to long-term, low-dose exposures, as well as to understand the broader implications of NPs in animal and human health [2].

The integration of nanotechnology into ruminant nutrition offers significant benefits, including enhanced nutrient bioavailability, improved feed efficiency, and potential reductions in antibiotic use. However, the unique physicochemical properties of nanomaterials—such as size, shape, surface area, and surface charge—also raise concerns regarding their safety for animals, humans, and the environment. To address these concerns, a comprehensive, multi-tiered safety validation framework is required.

*Physicochemical Characterization*: A thorough characterization of nanomaterials is the first step in ensuring reproducibility and safety. Key parameters include particle size, morphology, surface area, and surface charge. Techniques such as Dynamic Light Scattering, Transmission Electron Microscopy (TEM), and zeta potential analysis are commonly used to quantify these properties. This information is essential to predict interactions with biological systems, stability in feed matrices, and potential bioaccumulation [99].

In Vitro *Toxicological Assessment*: In vitro assays provide rapid, controlled screening of nanomaterial toxicity. Relevant tests include cytotoxicity assays (e.g., MTT, LDH release), genotoxicity assays (e.g., comet assay), and studies of oxidative stress or membrane permeability on cell lines such as rumen epithelial cells and hepatocytes [100]. These assays allow early detection of potential adverse effects, enabling optimization of nanomaterial design before in vivo testing.

In Vivo *Evaluation*: In vivo studies remain the gold standard for safety validation. Controlled feeding trials in target ruminant species assess the impact of nanomaterials on growth performance, nutrient absorption, gastrointestinal integrity, immune response, and tissue accumulation. Long-term, low-dose exposure studies are particularly important to understand chronic effects, as extrapolation from short-term, high-dose studies can be misleading. Measurements of bioaccumulation in organs, oxidative stress markers, and potential transfer into edible products (milk and meat) are critical to ensure animal health and food safety.

*Environmental and Occupational Safety*: Nanoparticles may enter the environment through excreta or feed processing, raising concerns about soil and water contamination, biodiversity impacts, and occupational exposure. Environmental risk assessments, along with proper handling protocols and protective measures for workers, are essential components of a responsible safety framework [101].

*Regulatory Compliance and Analytical Validation*: Regulatory bodies, including the European Food Safety Authority (EFSA) and U.S. Food and Drug Administration (FDA), emphasize validated analytical methods for detecting and quantifying nanomaterials in feed and food matrices [99]. This ensures compliance with evolving guidelines, informs permissible concentration limits, and supports labeling standards. Analytical methods may include spectroscopic, chromatographic, and microscopy-based techniques.

*Integrated Risk Assessment and Future Perspectives*: The safety evaluation of nanomaterials should integrate physicochemical characterization, in vitro and in vivo testing, environmental risk assessment, and regulatory compliance. Collaborative efforts among nanotechnologists, animal scientists, veterinarians, feed manufacturers, and regulatory agencies are essential to translate laboratory innovations into practical, scalable, and safe applications.

Advancements in nano-sensors, artificial intelligence, and precision livestock technologies may further support real-time monitoring of animal responses to nano-additives, enabling individualized nutrition strategies and early detection of adverse effects. By adhering to a robust safety validation framework, nanotechnology can be responsibly integrated into ruminant production, maximizing benefits while minimizing risks to animals, humans, and the environment.

## 8. Challenges, Limitations, Future Perspectives and Economic Considerations

Nanotechnology has made significant strides in advancing disease detection, prevention, and treatment in animals, although potential toxic side effects remain a concern [2,21]. The biological effects of engineered NPs may differ from naturally occurring ones due to variations in size, surface characteristics, and interactions with biological systems [102]. These particles are often designed with protective coatings to enhance bioavailability and functionality by evading the body’s defense mechanisms.

Many NPs-based products are incorporated into finished goods, limiting direct contact with animals or the environment. However, concerns persist regarding their potential release into ecosystems, occupational exposure, and broader ethical issues, including self-replicating nano-machines and transparency in marketing [103]. Comprehensive research is still needed to understand the long-term impacts of engineered NPs on animal health, human safety, and environmental sustainability [104].

Despite potential benefits, adoption of nanotechnology faces barriers such as high production costs, limited awareness, and regulatory uncertainties. As manufacturing becomes more efficient and standardized safety regulations are established, broader accessibility is expected, especially in resource-limited settings where efficient feed utilization is crucial. Regulatory bodies such as the FDA and EFSA will play a pivotal role in establishing safety guidelines for responsible use.

Replacing antibiotics with NPs-based alternatives remains a long-term goal, constrained by the need for extensive in vivo testing, food safety evaluation, and regulatory approval. Some applications, such as antiseptic wound dressings, have already been integrated practically. Future developments may include NPs with anticancer or immune-modulating effects, but their cytotoxicity must be carefully assessed in both healthy and diseased cells. Long-term studies are required to evaluate bioaccumulation in organs and impacts on rumen microbiota, digestive health, and overall animal performance.

Integration of nanotechnology with artificial intelligence, machine learning, and precision livestock farming could revolutionize nutritional strategies. For example, nano-sensors embedded in automated feeding systems could monitor feed intake, rumen function, and animal behavior in real time, enabling individualized precision feeding. AI models could also predict interactions between nano-materials and biological systems, accelerating the development of safe and effective nano-additives.

Current research in ruminant nutrition has focused primarily on nano-encapsulated probiotics and enzymes, but expanding into nano-minerals and nano-vaccines presents additional opportunities. Nano-minerals could improve nutrient bioavailability and support individualized nutrition strategies, while nano-vaccines could enhance antigen stability and delivery, strengthening immune defenses and potentially reducing antibiotic use. As antimicrobial resistance becomes an increasing concern, such innovations could provide essential tools for sustainable livestock production.

To ensure safe implementation, validated safety assessment methods—including in vitro cytotoxicity assays, in vivo feeding trials, pharmacokinetics, bioaccumulation monitoring, and advanced particle characterization—should be incorporated into regulatory frameworks and standard operating procedures. Potential risks to soil, water, biodiversity, and unintended effects on animal health require careful consideration.

Research gaps and future directions include:

*Long-term biosafety*: Comprehensive in vivo studies to assess chronic toxicity, bioaccumulation, and potential organ-specific effects.

*Interactions with rumen microbiota*: Studies to understand how NPs influence microbial diversity, fermentation efficiency, and nutrient utilization.

*Standardized testing protocols*: Development of harmonized in vitro and in vivo methods, including cytotoxicity assays, pharmacokinetics, and particle characterization, to support regulatory approval.

*Environmental impact assessment*: Research on NP release into soil, water, and ecosystems to minimize ecological risks.

*Regulatory and ethical frameworks*: Establishing clear guidelines on permissible NP concentrations, labeling standards, and ethical considerations for safe livestock application.

Collaborative research among nanotechnologists, animal scientists, veterinarians, and environmental experts is vital to advance the field. Partnerships with feed companies, biotechnology firms, and agricultural extension services can help translate laboratory innovations into practical, scalable solutions for farmers. These collaborations will drive sustainable innovations and facilitate the safe and efficient adoption of nanotechnology in livestock systems.

*Economic considerations*: The adoption of nanotechnology in livestock production will be influenced by economic factors. In developed countries, higher production costs may be offset by greater access to advanced infrastructure, regulatory support, and potential gains in productivity and feed efficiency. In contrast, developing countries may face more significant barriers due to limited resources, higher relative costs, and lower awareness of nanotechnology applications. Cost–benefit analyses and context-specific strategies are therefore essential to ensure equitable and sustainable implementation across diverse production systems.

## 9. Analytical Challenges in Nanotechnology Applications

Despite the promising benefits of NPs in ruminant nutrition, one critical barrier is the analytical assessment of these materials in biological systems. Characterizing NPs in feed, gastrointestinal fluids, tissues, and excreta is notoriously difficult due to their small size, tendency to aggregate, and complex interactions with biomolecules. Standard analytical protocols—developed for bulk materials—can be misleading: the unique physicochemical properties of NPs often interfere with conventional assays, leading to false positives or negatives [105].

Moreover, the feed production process itself (e.g., mixing, pelleting) can alter nanoparticle stability, causing aggregation or degradation that changes their behavior in vivo [28]. Once administered, NPs can acquire a “protein corona”—a layer of adsorbed proteins—that modifies their surface identity and biodistribution, complicating interpretation of uptake, accumulation, and toxicity [27].

From an analytical perspective, distinguishing engineered nanoparticles from the much larger background of naturally occurring or incidental nanoparticles remains a major challenge. Techniques such as inductively coupled plasma mass spectrometry (ICP-MS) can be highly sensitive, but they still require rigorous method validation to ensure that the detected signals truly correspond to the engineered forms rather than dissolved ions or naturally present particles. This was evident in studies using in vitro digestion models with ICP-MS to track nano-Ag in pig and chicken feed, where researchers implemented additional separation and calibration steps to differentiate nano-scale particulate silver (typically in the nanometer range) from ionic silver released during gastrointestinal digestion and to accurately quantify transformation processes during the assay [106].

Given these complexities, rigorous and standardized analytical frameworks are urgently needed. These should include not only physical-chemical characterization (size, charge, aggregation state) but also biologically relevant assessments (stability in feed, gastrointestinal transit behavior, biodistribution, accumulation, and excretion). Without such data, any claimed benefits of NP supplementation must be interpreted cautiously, and safety evaluations may remain superficial.

## 10. Conclusions

Nanotechnology is reshaping ruminant nutrition by improving nutrient utilization, supporting animal health, and helping lower environmental impacts such as methane emissions and mineral waste. These advances can translate into meaningful improvements in productivity and sustainability across livestock systems. Equally important, emerging economic evidence shows that nano-enabled feed strategies can offer a favorable input–output ratio. Although nano-additives often carry a higher upfront cost, their superior bioavailability and targeted delivery allow for reduced inclusion rates, better feed conversion, and improved animal performance—factors that together can offset initial expenses and enhance overall profitability for producers. This economic advantage reinforces the need for continued research and development in the field.

As manufacturing technologies advance and regulatory frameworks become more clearly defined, especially in developing regions, adoption is expected to expand. Transparent, evidence-based communication will be essential for building public trust and supporting responsible integration into production systems. Pairing nanotechnology with precision livestock tools promises even greater gains through real-time monitoring and individualized nutrition. Future work should address underexplored innovations such as nano-delivery systems for amino acids, vitamins, and organic acids, as well as the development of biologically synthesized nanoparticles that may offer more sustainable and cost-effective alternatives, provided their safety and efficacy are fully validated.

## Figures and Tables

**Table 1:** Sources and Applications of Nano-Feed Additives (In vivo and in vitro experiments).

Source	Additives	Application	References
Microorganisms	Probiotics	Enhancing digestive performance and supporting growth.	Zhao et al. [25], Muzaffar et al. [26]
Minerals	Functional elements and NPs	Synergistic antimicrobial activity, improved antioxidant capacity, and optimized digestive efficiency.	Abdelnour et al. [27]
Essential Oils	Phytogenic additives	Antimicrobial properties, enhanced digestive function, and promotion of gut health.	Movahedi et al. [28]
Enzymes	Enzymes	Improved digestive function accompanied by antibacterial and antioxidant effects.	Pundir [8]
Oligosaccharides	Prebiotics	Enhanced digestive efficiency and promoted growth performance.	Muzaffar et al. [26]

**Table 2:** Effects of some nano-minerals on ruminant performance.

Additive and Doses	Study	Results	References
ZnO-NPs administered at concentrations of 100 and 200 mg/kg	In vitro fermentation	Enhanced the concentrations of volatile fatty acids (VFAs), microbial crude protein synthesis, and the fermentation efficiency of organic matter (OM). Negatively affected ammonia nitrogen (NH_3_-N) levels and reduced the acetate-to-propionate ratio. Enhanced the in vitro growth of ruminal microorganisms, boosted ruminal microbial protein synthesis, and improved energy utilization efficiency.	Chen et al. [31]
60 ppm of nano-Zn	Lactating crossbred Holstein Friesian cow	Significant reduction in somatic cell count. Increase in milk production.	Rajendran et al. [32]
0.5, 1.0 and 2.0 µg nano-Zn/mL semen	Buffalo	Elimination of damaged spermatozoa. Improved semen quality and enhanced the effectiveness of artificial insemination.	Bisla et al. [33]
1, 3, 5, 10, 20, 30, 40, and 50 mg nano-Zn/mL milk	Cows with clinical mastitis	Effectively inhibited the growth of bacteria, molds, and yeasts in buffaloes.	Hozyen et al. [34]
Nano-Selenium (nano-Se) at 0, 1.5, 3, and 4.5 ppm	In vitro fermentation	Did not affect the pH of rumen fluid and total or partial VFA concentrations. Higher concentrations of nano-Se additives enhanced the in vitro digestibility of dry matter (DM). Total gas and CH_4_ production decreased as the nano-Se levels increased.	González-Lemus et al. [35]
Nano-Se at 0, 0.1, 0.3, and 0.5 mg/Kg of DM	Holstein dairy cows	Linear increase in total VFA concentrations and the molar ratios of propionate and butyrate. Decreased rumen pH, NH_3_-N concentration, and the acetate to propionate ratio. The 0.3 mg/kg treatment group exhibited higher propionate levels. Quadratic improvements in the digestibility of DM, OM, crude protein (CP), neutral detergent fiber (NDF), and acid detergent fiber (ADF). Linear increase in total Se absorption.	Wei et al. [36]

**Table 3:** Current Status and Applications of Nanoparticle-Based Sensors in Ruminant Production.

Sensor Type	Nanomaterial/Technology	Application in Livestock/Feed	Current Status/Strengths
Quantum Dot-based sensors	Quantum dots (e.g., Cd-free, carbon)	Mycotoxin detection (e.g., aflatoxin), contaminated feed	High sensitivity, tunable fluorescence, rapid on-site detection
Nanozyme/Aptamer Sensors	Au-NPs, nanozymes (e.g., peroxidase-mimicking), aptamer-functionalized NPs	Detection of antibiotic residues, hormones, mycotoxins in feed or animal fluids	Selectivity vs. complex biological samples, scale-up, integration into farm systems
Electrochemical/Implantable Nano-sensors	Magnetic NPs, electrochemical nano-biosensors	In vivo monitoring of pH, glucose, metabolic biomarkers in ruminants	Long-term stability in vivo, biocompatibility, surgical implantation challenges
Nanofiber-enhanced surface-enhanced Raman scattering (SERS) Sensors	Electrospun nanofibers decorated with Au-NPs or Ag-NPs	Detection of contaminants (e.g., antibiotics, hormone residues) in meat or feed	Sampling from live animals, miniaturization, field validation in livestock systems

## Data Availability

No data was used for the research described in the article.

## Abbreviations

The following abbreviations are used in this manuscript:

ADF	Acid Detergent Fiber
CH_4_	Methane
CP	Crude Protein
DM	Dry Matter
DNA	Deoxyribonucleic Acid
EFSA	European Food Safety Authority
FDA	Food and Drug Administration
GIT	Gastrointestinal Tract
NDF	Neutral Detergent Fiber
NH_3_	Ammonia
NP	Nano-particle
OM	Organic Matter
PDMAEMA	Poly(2-dimethylamino)ethyl Methacrylate
ROS	Reactive Oxygen Species
VFA	Volatile Fatty Acids

## References

[1] Hill E.K., Li J. (2017). Current and Future Prospects for Nanotechnology in Animal Production. J. Anim. Sci. Biotechnol..

[2] Gelaye Y. (2024). Application of Nanotechnology in Animal Nutrition: Bibliographic Review. Cogent Food Agric..

[3] Fesseha H., Degu T., Getachew Y. (2020). Nanotechnology and Its Application in Animal Production: A Review. Vet. Med. Open J..

[4] Das A., Adhikari S., Deka D., Baildya N., Sahare P., Banerjee A., Paul S., Bisgin A., Pathak S. (2023). An Updated Review on the Role of Nanoformulated Phytochemicals in Colorectal Cancer. Medicina.

[5] Han C.S., Kaur U., Bai H., Roqueto dos Reis B., White R., Nawrocki R.A., Voyles R.M., Kang M.G., Priya S. (2022). Invited Review: Sensor Technologies for Real-Time Monitoring of the Rumen Environment. J. Dairy Sci..

[6] Kazemi M. (2025). Revolutionizing Veterinary Medicine: The Role of Nanoparticles in Advancing Animal Health, Nutrition and Disease Management. Vet. Med. Sci..

[7] Michalak I., Dziergowska K., Alagawany M., Farag M.R., El-Shall N.A., Tuli H.S., Emran T.B., Dhama K. (2022). The Effect of Metal-Containing Nanoparticles on the Health, Performance and Production of Livestock Animals and Poultry. Vet. Q..

[8] Pundir C.S. (2015). Enzyme Nanoparticles.

[9] Iavicoli I., Leso V., Beezhold D.H., Shvedova A.A. (2017). Nanotechnology in Agriculture: Opportunities, Toxicological Implications, and Occupational Risks. Toxicol. Appl. Pharmacol..

[10] Liberati A., Altman D.G., Tetzlaff J., Mulrow C., Gøtzsche P.C., Ioannidis J.P.A., Clarke M., Devereaux P.J., Kleijnen J., Moher D. (2009). The PRISMA Statement for Reporting Systematic Reviews and Meta-Analyses of Studies That Evaluate Healthcare Interventions: Explanation and Elaboration. BMJ.

[11] Moher D., Liberati A., Tetzlaff J., Altman D.G., Altman D., Antes G., Atkins D., Barbour V., Barrowman N., Berlin J.A. (2009). Preferred Reporting Items for Systematic Reviews and Meta-Analyses: The PRISMA Statement. PLoS Med..

[12] Jagtiani E. (2022). Advancements in Nanotechnology for Food Science and Industry. Food Front..

[13] Chin S., Moniruzzaman M., Smirnova E., Thoung D.T.C., Sureshbabu A., Karthikeyan A., Lee D.I., Min T. (2025). Green Metal Nanotechnology in Monogastric Animal Health: Current Trends and Future Prospects—A Review. Anim. Biosci..

[14] Perveen I., Saleem N., Nazir S., Nimra A., Siddiqui M.F., Alvi F.N., Koser N., Hussain W., Kiran A., Sajjad N. (2024). Nano-Feed Additives in Animal Nutrition, Their Preparation, Mode of Action and Application. Complementary and Alternative Medicine: Feed Additives.

[15] Sawant A., Kamath S., KG H., Kulyadi G.P. (2021). Solid-in-Oil-in-Water Emulsion: An Innovative Paradigm to Improve Drug Stability and Biological Activity. AAPS PharmSciTech.

[16] Schröder H.C., Neufurth M., Zhou H., Wang S., Wang X., Müller W.E.G. (2022). Inorganic Polyphosphate: Coacervate Formation and Functional Significance in Nanomedical Applications. Int. J. Nanomed..

[17] Vehring R., Snyder H., Lechuga-Ballesteros D. (2020). Spray Drying. Drying Technologies for Biotechnology and Pharmaceutical Applications.

[18] Joudeh N., Linke D. (2022). Nanoparticle Classification, Physicochemical Properties, Characterization, and Applications: A Comprehensive Review for Biologists. J. Nanobiotechnol..

[19] Qadeer A., Khan A., Khan N.M., Wajid A., Ullah K., Skalickova S., Chilala P., Slama P., Horky P., Alqahtani M.S. (2024). Use of Nanotechnology-Based Nanomaterial as a Substitute for Antibiotics in Monogastric Animals. Heliyon.

[20] Gopi M., Pearlin B., Kumar R.D., Shanmathy M., Prabakar G. (2017). Role of Nanoparticles in Animal and Poultry Nutrition: Modes of Action and Applications in Formulating Feed Additives and Food Processing. Int. J. Pharmacol..

[21] Almeida C.F., Faria M., Carvalho J., Pinho E. (2024). Contribution of Nanotechnology to Greater Efficiency in Animal Nutrition and Production. J. Anim. Physiol. Anim. Nutr..

[22] Ghazy O.A., Fouad M.T., Morsy T.A., Kholif A.E. (2023). Nanoemulsion Formulation of *Lawsonia inermis* Extract and Its Potential Antimicrobial and Preservative Efficacy against Foodborne Pathogens. Food Control.

[23] Ghazy O.A., Fouad M.T., Saleh H.H., Kholif A.E., Morsy T.A. (2021). Ultrasound-Assisted Preparation of Anise Extract Nanoemulsion and Its Bioactivity against Different Pathogenic Bacteria. Food Chem..

[24] Seven P.T., Seven I., Gul Baykalir B., Iflazoglu Mutlu S., Salem A.Z.M. (2018). Nanotechnology and Nano-Propolis in Animal Production and Health: An Overview. Ital. J. Anim. Sci..

[25] Zhao C., Zhu Y., Kong B., Huang Y., Yan D., Tan H., Shang L. (2020). Dual-Core Prebiotic Microcapsule Encapsulating Probiotics for Metabolic Syndrome. ACS Appl. Mater. Interfaces.

[26] Muzaffar K., Jan R., Ahmad Bhat N., Gani A., Ahmed Shagoo M. (2021). Commercially Available Probiotics and Prebiotics Used in Human and Animal Nutrition. Advances in Probiotics.

[27] Abdelnour S.A., Alagawany M., Hashem N.M., Farag M.R., Alghamdi E.S., Hassan F.U., Bilal R.M., Elnesr S.S., Dawood M.A.O., Nagadi S.A. (2021). Nanominerals: Fabrication Methods, Benefits and Hazards, and Their Applications in Ruminants with Special Reference to Selenium and Zinc Nanoparticles. Animals.

[28] Movahedi F., Nirmal N., Wang P., Jin H., Grøndahl L., Li L. (2024). Recent Advances in Essential Oils and Their Nanoformulations for Poultry Feed. J. Anim. Sci. Biotechnol..

[29] Byrne L., Murphy R.A. (2022). Relative Bioavailability of Trace Minerals in Production Animal Nutrition: A Review. Animals.

[30] Schmidt C.W. (2009). Nanotechnology-Related Environment, Health, and Safety Research: Examining the National Strategy. Environ. Health Perspect..

[31] Chen J., Wang W., Wang Z. (2011). Effect of Nano-Zinc Oxide Supplementation on Rumen Fermentation In Vitro. Chin. J. Anim. Nutr..

[32] Rajendran D., Kumar G., Ramakrishnan S., Shibi T.K. (2013). Enhancing the Milk Production and Immunity in Holstein Friesian Crossbred Cow by Supplementing Novel Nano Zinc Oxide. Res. J. Biotechnol..

[33] Bisla A., Rautela R., Yadav V., Singh P., Kumar A., Ghosh S., Kumar A., Bag S., Kumar B., Srivastava N. (2020). Nano-Purification of Raw Semen Minimises Oxidative Stress with Improvement in Post-Thaw Quality of Buffalo Spermatozoa. Andrologia.

[34] Hozyen H.F., Ibrahim E.S., Khairy E.A., El-Dek S.I. (2019). Enhanced Antibacterial Activity of Capped Zinc Oxide Nanoparticles: A Step towards the Control of Clinical Bovine Mastitis. Vet. World.

[35] González-Lemus U., Medina-Pérez G., Peláez-Acero A., Campos-Montiel R.G. (2022). Decrease of Greenhouse Gases during an In Vitro Ruminal Digestibility Test of Forage (*Festuca arundinacea*) Conditioned with Selenium Nanoparticles. Nanomaterials.

[36] Wei J.Y., Wang J., Liu W., Zhang K.Z., Sun P. (2019). Short Communication: Effects of Different Selenium Supplements on Rumen Fermentation and Apparent Nutrient and Selenium Digestibility of Mid-Lactation Dairy Cows. J. Dairy Sci..

[37] Rahman H.S., Othman H.H., Abdullah R., Edin H.Y.A.S., AL-Haj N.A. (2022). Beneficial and Toxicological Aspects of Zinc Oxide Nanoparticles in Animals. Vet. Med. Sci..

[38] Sitaresmi P., Hudaya M., Atmoko B., Wulandari W., Ujilestari T. (2024). Production and Effects of Nanomineral Selenium (Nano-Se) Feed Additive on Rumen Fermentation, Productivity, and Reproductive Performance of Ruminants. J. Adv. Vet. Anim. Res..

[39] Khazraei S.K., Tabeidian S.A., Habibian M. (2022). Selenium Nanoparticles Are More Efficient than Sodium Selenite in Reducing the Toxicity of Aflatoxin B1 in Japanese Quail. Vet. Med. Sci..

[40] Yarmohammadi A.B., Sharifi S.D., Mohammadi-Sangcheshmeh A. (2020). Efficacy of Dietary Supplementation of Nanoparticles-Chromium, Chromium-Methionine and Zinc-Proteinate, on Performance of Japanese Quail under Physiological Stress. Ital. J. Anim. Sci..

[41] Ahmed J., Vasagam K.P.K., Ramalingam K. (2023). Nanoencapsulated Aquafeeds and Current Uses in Fisheries/Shrimps: A Review. Appl. Biochem. Biotechnol..

[42] Iommelli P., Spina A.A., Vastolo A., Infascelli L., Lotito D., Musco N., Tudisco R. (2025). Functional and Economic Role of Some Mediterranean Medicinal Plants in Dairy Ruminants’ Feeding: A Review of the Effects of Garlic, Oregano, and Rosemary. Animals.

[43] Kholif A.E., Olafadehan O.L., Kholif A.M.M., Ghavipanje N., Vargas-Bello-Pérez E., Anele U.Y. The Role of Encapsulated Essential Oils in Reducing Methane Production from Ruminant Animals—A Review. Ann. Anim. Sci..

[44] Kholif A.E., Olafadehan O.A. (2021). Essential Oils and Phytogenic Feed Additives in Ruminant Diet: Chemistry, Ruminal Microbiota and Fermentation, Feed Utilization and Productive Performance. Phytochem. Rev..

[45] Hassan F.U., Arshad M.A., Ebeid H.M., Rehman M.S.-U., Khan M.S., Shahid S., Yang C. (2020). Phytogenic Additives Can Modulate Rumen Microbiome to Mediate Fermentation Kinetics and Methanogenesis through Exploiting Diet–Microbe Interaction. Front. Vet. Sci..

[46] Wang L., Hu C., Shao L. (2017). The Antimicrobial Activity of Nanoparticles: Present Situation and Prospects for the Future. Int. J. Nanomed..

[47] Sarwar S., Chakraborti S., Bera S., Sheikh I.A., Hoque K.M., Chakrabarti P. (2016). The Antimicrobial Activity of ZnO Nanoparticles against *Vibrio cholerae*: Variation in Response Depends on Biotype. Nanomedicine.

[48] Sarker N.C., Keomanivong F., Borhan M., Rahman S., Swanson K. (2018). In Vitro Evaluation of Nano Zinc Oxide (NZnO) on Mitigation of Gaseous Emissions. J. Anim. Sci. Technol..

[49] Xie S., Ying Z., Xiu Z., Sun Y., Yang Q., Gao H., Fan W., Wu Y. (2024). Zinc Oxide Nanoparticles Improve Lactation and Metabolism in Dairy Goats by Modulating the Rumen Microbiota. Front. Microbiol..

[50] Petrič D., Mikulová K., Bombárová A., Batťányi D., Čobanová K., Kopel P., Łukomska A., Pawlak P., Sidoruk P., Kotwica S. (2024). Efficacy of Zinc Nanoparticle Supplementation on Ruminal Environment in Lambs. BMC Vet. Res..

[51] Altermann E., Reilly K., Young W., Ronimus R.S., Muetzel S. (2022). Tailored Nanoparticles with the Potential to Reduce Ruminant Methane Emissions. Front. Microbiol..

[52] Dawood T.N. (2025). The Effect of Beetroot Extract with Silver Nano Particles on Rumen Parameters in Awassi Lambs. Maced. Vet. Rev..

[53] Albuquerque J., Casal S., Páscoa R.N.M.J., Van Dorpe I., Fonseca A.J.M., Cabrita A.R.J., Neves A.R., Reis S. (2020). Applying Nanotechnology to Increase the Rumen Protection of Amino Acids in Dairy Cows. Sci. Rep..

[54] Gupta A., Mumtaz S., Li C.-H., Hussain I., Rotello V.M. (2019). Combatting Antibiotic-Resistant Bacteria Using Nanomaterials. Chem. Soc. Rev..

[55] Zou L., Wang J., Gao Y., Ren X., Rottenberg M.E., Lu J., Holmgren A. (2018). Synergistic Antibacterial Activity of Silver with Antibiotics Correlating with the Upregulation of the ROS Production. Sci. Rep..

[56] Shikha S., Chaudhuri S.R., Bhattacharyya M.S. (2020). Facile One Pot Greener Synthesis of Sophorolipid Capped Gold Nanoparticles and Its Antimicrobial Activity Having Special Efficacy Against Gram Negative Vibrio Cholerae. Sci. Rep..

[57] Khater M.S., Kulkarni G.R., Khater S.S., Gholap H., Patil R. (2020). Study to Elucidate Effect of Titanium Dioxide Nanoparticles on Bacterial Membrane Potential and Membrane Permeability. Mater. Res. Express.

[58] Awuchi C.G., Ondari E.N., Ogbonna C.U., Upadhyay A.K., Baran K., Okpala C.O.R., Korzeniowska M., Guiné R.P.F. (2021). Mycotoxins Affecting Animals, Foods, Humans and Plants: Types, Occurrence, Toxicities, Action Mechanisms, Prevention and Detoxification Strategies-a Revisit. Foods.

[59] Chuai Q., Zhu X., An Y., Liu H., Sun B. (2025). Advances in Optical Sensor Visualization: Enabling Rapid Mycotoxin Detection. Trends Food Sci. Technol..

[60] Dabare S., Rajapaksha S., Munaweera I. (2025). Empowering Innovative Strategies: Utilizing Polymer-Based Nanotechnology for the Prevention, Control, and Detection of Aflatoxins, Ochratoxins, and Fusarium Toxins in Food Systems. Grain Oil Sci. Technol..

[61] Gholami-Ahangaran M., Zia-Jahromi N. (2013). Nanosilver Effects on Growth Parameters in Experimental Aflatoxicosis in Broiler Chickens. Toxicol. Ind. Health.

[62] Jiang Y., Chu N., Zeng R.J. (2019). Submersible Probe Type Microbial Electrochemical Sensor for Volatile Fatty Acids Monitoring in the Anaerobic Digestion Process. J. Clean. Prod..

[63] Khizar S., Elaissari A., Al-Dossary A.A., Zine N., Jaffrezic-Renault N., Errachid A. (2022). Advancement in Nanoparticle-Based Biosensors for Point-of-Care In Vitro Diagnostics. Curr. Top. Med. Chem..

[64] De Anda-Flores Y., Carvajal-Millan E., Campa-Mada A., Lizardi-Mendoza J., Rascon-Chu A., Tanori-Cordova J., Martínez-López A.L. (2021). Polysaccharide-Based Nanoparticles for Colon-Targeted Drug Delivery Systems. Polysaccharides.

[65] Zandanel C., Ponchel G., Noiray M., Vauthier C. (2021). Nanoparticles Facing the Gut Barrier: Retention or Mucosal Absorption? Mechanisms and Dependency to Nanoparticle Characteristics. Int. J. Pharm..

[66] Zheng G., Zhang B., Yu H., Song Z., Xu X., Zheng Z., Zhao K., Zhao J., Zhao Y. (2025). Therapeutic Applications and Potential Biological Barriers of Nano-Delivery Systems in Common Gastrointestinal Disorders: A Comprehensive Review. Adv. Compos. Hybrid Mater..

[67] Qiao X., Bao L., Liu G., Cui X. (2024). Nanomaterial Journey in the Gut: From Intestinal Mucosal Interaction to Systemic Transport. Nanoscale.

[68] Vitulo M., Gnodi E., Meneveri R., Barisani D. (2022). Interactions between Nanoparticles and Intestine. Int. J. Mol. Sci..

[69] Sinnecker H., Krause T., Koelling S., Lautenschläger I., Frey A. (2014). The Gut Wall Provides an Effective Barrier against Nanoparticle Uptake. Beilstein J. Nanotechnol..

[70] Hewa Nadugala B., Pagel C.N., Raynes J.K., Ranadheera C.S., Logan A. (2022). The Effect of Casein Genetic Variants, Glycosylation and Phosphorylation on Bovine Milk Protein Structure, Technological Properties, Nutrition and Product Manufacture. Int. Dairy J..

[71] Kumari A., Chauhan A.K. (2022). Iron Nanoparticles as a Promising Compound for Food Fortification in Iron Deficiency Anemia: A Review. J. Food Sci. Technol..

[72] Sagar N.A., Kumar N., Choudhary R., Bajpai V.K., Cao H., Shukla S., Pareek S. (2022). Prospecting the Role of Nanotechnology in Extending the Shelf-Life of Fresh Produce and in Developing Advanced Packaging. Food Packag. Shelf Life.

[73] Muslykhah U., Phupaboon S., Suriyapha C., Sommai S., Pongsub S., Dagaew G., Matra M., Wanapat M. (2024). Effects of Phytonutrient-Based Encapsulation of Wolffia Globosa on Gas Production, In Vitro Fermentation Characteristics, and Methane Mitigation Using In Vitro Study Techniques. Ital. J. Anim. Sci..

[74] El-Nile A.E., Elazab M.A., Soltan Y.A., Elkomy A.E., El-Zaiat H.M., Sallam S.M.A., El-Azrak K.E.-D. (2023). Nano and Natural Zeolite Feed Supplements for Dairy Goats: Feed Intake, Ruminal Fermentation, Blood Metabolites, and Milk Yield and Fatty Acids Profile. Anim. Feed Sci. Technol..

[75] Swain P.S., Rao S.B.N., Rajendran D., Dominic G., Selvaraju S. (2016). Nano Zinc, an Alternative to Conventional Zinc as Animal Feed Supplement: A Review. Anim. Nutr..

[76] Waters S.M., Roskam E., Smith P.E., Kenny D.A., Popova M., Eugène M., Morgavi D.P. (2025). The Role of Rumen Microbiome in the Development of Methane Mitigation Strategies for Ruminant Livestock. J. Dairy Sci..

[77] Kholif A.E. (2024). The Impact of Varying Levels of *Laurus nobilis* Leaves as a Sustainable Feed Additive on Ruminal Fermentation: In Vitro Gas Production, Methane and Carbon Dioxide Emissions, and Ruminal Degradability of a Conventional Diet for Ruminants. Fermentation.

[78] Alabi J.O., Wuaku M., Anotaenwere C.C., Okedoyin D.O., Adelusi O.O., Ike K.A., Gray D., Kholif A.E., Subedi K., Anele U.Y. (2024). A Mixture of Prebiotics, Essential Oil Blends, and Onion Peel Did Not Affect Greenhouse Gas Emissions or Nutrient Degradability, but Altered Volatile Fatty Acids Production in Dairy Cows Using Rumen Simulation Technique (RUSITEC). Fermentation.

[79] Wang R., Bin S.H., Wang M., Lin B., Deng J.P., Tan L.W., Liu W.X., Sun X.Z., Teklebrhan T., Tan Z.L. (2019). Effects of Elemental Magnesium and Magnesium Oxide on Hydrogen, Methane and Volatile Fatty Acids Production in In Vitro Rumen Batch Cultures. Anim. Feed Sci. Technol..

[80] Hernández-Sánchez D., Cervantes-Gómez D., Ramírez-Bribiesca J.E., Cobos-Peralta M., Pinto-Ruiz R., Astigarraga L., Gere J.I. (2019). The Influence of Copper Levels on In Vitro Ruminal Fermentation, Bacterial Growth and Methane Production. J. Sci. Food Agric..

[81] Jiang Q., Liu H., Zhang Y., Cui M., Fu B., Liu H. (2021). Insight into Sludge Anaerobic Digestion with Granular Activated Carbon Addition: Methanogenic Acceleration and Methane Reduction Relief. Bioresour. Technol..

[82] Sung Y.J., Suk H.J., Sung H.Y., Li T., Poo H., Kim M.G. (2013). Novel Antibody/Gold Nanoparticle/Magnetic Nanoparticle Nanocomposites for Immunomagnetic Separation and Rapid Colorimetric Detection of Staphylococcus Aureus in Milk. Biosens. Bioelectron..

[83] Lee Y.-K., Jung S.K., Chang Y.H., Kwak H.-S. (2017). Highly Bioavailable Nanocalcium from Oyster Shell for Preventing Osteoporosis in Rats. Int. J. Food Sci. Nutr..

[84] Lee Y.K., Ahn S.I., Chang Y.H., Kwak H.S. (2015). Physicochemical and Sensory Properties of Milk Supplemented with Dispersible Nanopowdered Oyster Shell during Storage. J. Dairy Sci..

[85] Madakka M., Rajesh N., Rajeswari J. (2020). Immunocomposition of Gastrointestinal Tract of Gut. Immunotherapy for Gastrointestinal Malignancies.

[86] Fubini B., Ghiazza M., Fenoglio I. (2010). Physico-Chemical Features of Engineered Nanoparticles Relevant to Their Toxicity. Nanotoxicology.

[87] Sun Y., Kinsela A.S., Cen X., Sun S., Collins R.N., Cliff D.I., Wu Y., Waite T.D. (2022). Impact of Reactive Iron in Coal Mine Dust on Oxidant Generation and Epithelial Lung Cell Viability. Sci. Total Environ..

[88] Liu A.A., Henin S., Abbaspoor S., Bragin A., Buffalo E.A., Farrell J.S., Foster D.J., Frank L.M., Gedankien T., Gotman J. (2022). A Consensus Statement on Detection of Hippocampal Sharp Wave Ripples and Differentiation from Other Fast Oscillations. Nat. Commun..

[89] Eenennaam A.L. (2025). Van Current and Future Uses of Genetic Improvement Technologies in Livestock Breeding Programs. Anim. Front..

[90] Odhiambo J.F., DeJarnette J.M., Geary T.W., Kennedy C.E., Suarez S.S., Sutovsky M., Sutovsky P. (2014). Increased Conception Rates in Beef Cattle Inseminated with Nanopurified Bull Semen1. Biol. Reprod..

[91] Sarıözkan S., Bucak M.N., Tuncer P.B., Büyükleblebici S., Eken A., Akay C. (2015). Influence of Fetuin and Hyaluronan on the Post-Thaw Quality and Fertilizing Ability of Holstein Bull Semen. Cryobiology.

[92] Barkalina N., Jones C., Kashir J., Coote S., Huang X., Morrison R., Townley H., Coward K. (2014). Effects of Mesoporous Silica Nanoparticles upon the Function of Mammalian Sperm In Vitro. Nanomedicine.

[93] Agarwal S., Zhang Y., Maji S., Greiner A. (2012). PDMAEMA Based Gene Delivery Materials. Mater. Today.

[94] Pawar K., Kaul G. (2014). Toxicity of Titanium Oxide Nanoparticles Causes Functionality and DNA Damage in Buffalo (*Bubalus bubalis*) Sperm In Vitro. Toxicol. Ind. Health.

[95] Yip Y.J., Lee S.S.C., Neo M.L., Teo S.L.-M., Valiyaveettil S. (2022). A Comparative Investigation of Toxicity of Three Polymer Nanoparticles on Acorn Barnacle (*Amphibalanus amphitrite*). Sci. Total Environ..

[96] Yan X., Pan Z., Chen S., Shi N., Bai T., Dong L., Zhou D., White J.C., Zhao L. (2022). Rice Exposure to Silver Nanoparticles in a Life Cycle Study: Effect of Dose Responses on Grain Metabolomic Profile, Yield, and Soil Bacteria. Environ. Sci. Nano.

[97] Javed R., Ain N.U., Gul A., Arslan Ahmad M., Guo W., Ao Q., Tian S. (2022). Diverse Biotechnological Applications of Multifunctional Titanium Dioxide Nanoparticles: An up-to-Date Review. IET Nanobiotechnol..

[98] Wang L., Mello D.F., Zucker R.M., Rivera N.A., Rogers N.M.K., Geitner N.K., Boyes W.K., Wiesner M.R., Hsu-Kim H., Meyer J.N. (2021). Lack of Detectable Direct Effects of Silver and Silver Nanoparticles on Mitochondria in Mouse Hepatocytes. Environ. Sci. Technol..

[99] More S., Bampidis V., Benford D., Bragard C., Halldorsson T., Hernández-Jerez A., Hougaard Bennekou S., Koutsoumanis K., Lambré C., Machera K. (2021). Guidance on Risk Assessment of Nanomaterials to Be Applied in the Food and Feed Chain: Human and Animal Health. EFSA J..

[100] Kroll A., Pillukat M.H., Hahn D., Schnekenburger J. (2012). Interference of Engineered Nanoparticles with In Vitro Toxicity Assays. Arch. Toxicol..

[101] Quik J.T.K., Meesters J.A.J., Peijnenburg W.J.G.M., Brand W., Bleeker E.A.J. (2020). Environmental Risk Assessment (ERA) of the Application of Nanoscience and Nanotechnology in the Food and Feed Chain. EFSA Support. Publ..

[102] Liu W., Huang Y. (2022). Cell Membrane-Engineered Nanoparticles for Cancer Therapy. J. Mater. Chem. B.

[103] Hemathilake D.M.K.S., Gunathilake D.M.C.C., Bhat R. (2022). Agricultural Productivity and Food Supply to Meet Increased Demands. Future Foods.

[104] Liu W., Worms I.A.M., Jakšić Ž., Slaveykova V.I. (2022). Aquatic Organisms Modulate the Bioreactivity of Engineered Nanoparticles: Focus on Biomolecular Corona. Front. Toxicol..

[105] McNeil S.E. (2011). Challenges for Nanoparticle Characterization. Characterization of Nanoparticles Intended for Drug Delivery.

[106] Ben-Jeddou K., Bakir M., Jiménez M.S., Gómez M.T., Abad-Álvaro I., Laborda F. (2024). Nanosilver-Based Materials as Feed Additives: Evaluation of Their Transformations along In Vitro Gastrointestinal Digestion in Pigs and Chickens by Using an ICP-MS Based Analytical Platform. Anal. Bioanal. Chem..

